# Motion perception and eye movements predict reading readiness: Evidence from pre-readers in an action video game task

**DOI:** 10.1016/j.isci.2026.117063

**Published:** 2026-08-06

**Authors:** Niloufar Chamani, Samy Rima, Michael Christoph Schmid

**Affiliations:** 1 Department of Neuro- and Movement Sciences, Faculty of Science and Medicine, University of Fribourg, 1700 Fribourg, Switzerland; 2 Biosciences Institute, Newcastle University, Newcastle, UK

**Keywords:** developmental dyslexia, motion perception, eye movement control, rapid automatized naming, hemispheric lateralization, early reading skills

## Abstract

Developmental dyslexia (DD) is associated with deficits in visual motion perception and eye movement control, yet little is known about these processes at the pre-reading stage. We investigated motion perception and eye movement behavior in thirty-four pre-reading children (aged 4–6 years) during action video game Fruit Ninja (FN) play and rapid automatized naming (RAN) performance. FN and RAN scores were significantly correlated (r = 0.54, *p* = 0.001), suggesting a link between motion perception and object naming ability. While peripheral vision enabled faster fruit slicing (t = 11.28, *p* < 0.001), foveating objects was more predictive of FN and RAN performance. Children exhibited shorter reaction times for detecting right visual field (RVF) objects (t = 3.22, *p* = 0.003), aligning with adult findings. These results highlight the role of motion perception in reading development and support the potential of motion-based interventions for early reading difficulties.

## Highlights

• Action video game performance predicts reading readiness in pre-readers
• Efficient foveation underlies both action video game performance and rapid naming ability
• Visual lateralization precedes formal reading instruction

## Graphical abstract

**Figure:**
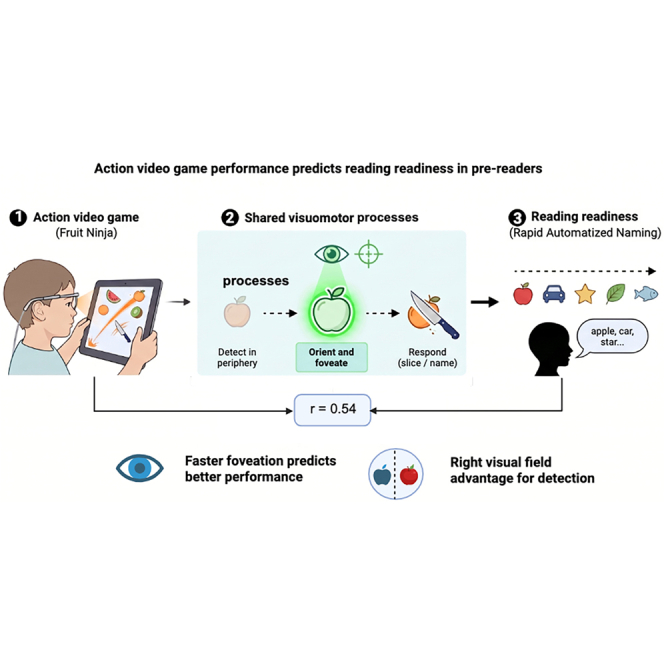

## Teaser

Natural sciences; Biological sciences; Neuroscience; Sensory neuroscience; Cognitive neuroscience

## Introduction

Reading is a hallmark of human cognition that depends on the dynamic interplay of multiple neural systems, including those supporting language processing, visual perception, attention, and oculomotor control. Despite this complexity, current diagnostic and intervention strategies for developmental dyslexia (DD) have focused predominantly on phonological and linguistic deficits, with considerably less emphasis on perceptual and attentional processes. However, a growing body of research suggests that non-verbal cognitive functions—particularly visual motion perception and attentional control—may play a foundational role in reading acquisition, even prior to formal reading instruction.

One way to assess reading skills is through rapid automatized naming (RAN), a task that measures the ability to quickly name a series of visual objects. RAN is significantly linked to reading fluency, with poor RAN performance frequently observed in individuals who struggle with reading.^1^ Notably, individuals with DD perform significantly slower on RAN tasks than typical readers,^2^ suggesting shared mechanisms between object recognition and naming speed and reading ability.

The RAN task is widely recognized as a meaningful early predictor of reading acquisition.^3^^,^^4^^,^^5^^,^^6^^,^^7^ Furthermore, longitudinal studies demonstrated that RAN speed of pre-readers predicts later reading proficiency.^3^^,^^5^^,^^8^^,^^9^

The predictive strength of the RAN task is robustly confirmed across different languages, providing significant evidence for its role as a universal marker of DD.^10^^,^^11^^,^^12^^,^^13^^,^^14^ In highly consistent or shallow orthographies, such as German, RAN speed deficits are consistently documented in children with DD,^3^^,^^4^^,^^15^^,^^16^^,^^17^^,^^18^^,^^19^^,^^20^ where studies comparing regular and irregular languages often find that difficulty lies primarily with reading speed (RS) rather than accuracy, leading to the designation of “speed dyslexia.”^16^^,^^18^^,^^19^^,^^20^ Previous research focusing on word recognition deficits in German confirmed RAN speed as a significant predictor of reading ability.^4^^,^^15^^,^^17^ Conversely, French is generally considered a more opaque or inconsistent alphabetic orthography compared with German,^21^^,^^22^ yet longitudinal research similarly confirms the predictive importance of RAN in French reading acquisition.^3^^,^^4^ This cross-linguistic reliability is substantiated by meta-analyses showing a systematic difference in RAN performance between non-impaired and dyslexic readers across both shallow and deep orthographies.^10^^,^^11^ Furthermore, a comprehensive longitudinal study, which investigated RAN (alongside phonological awareness) across five alphabetic orthographies including German and French, specifically concluded that RAN measures tap a universal mechanism that holds similar relevance in learning to read across these varied orthographies, irrespective of their complexity.^3^^,^^4^ This body of evidence, including cross-sectional studies examining RAN’s relationship with reading and spelling in first-grade French-speaking children,^22^^,^^23^ supports the conclusion that RAN consistently predicts reading skills across languages and serves as a reliable marker for DD across diverse linguistic systems.^11^

Given that RAN itself is considered a “microcosm of reading,” demanding fast visuo-verbal integration, lexical access, and attentional scanning, its early assessment is crucial for identifying children at risk for DD.^6^^,^^7^^,^^14^ This critical link has been further strengthened by experimental studies that manipulate and enhance RAN performance, establishing a causal pathway to reading improvement.^6^^,^^24^ For instance, studies using intensive, self-adaptive RAN training (RANt) software, which accelerates naming speed of serially presented visual stimuli by progressively reducing presentation time, led to significant improvements in reading decoding speed and accuracy in children with DD.^6^^,^^24^^,^^25^ Crucially, the gains obtained by exercising RAN alone were comparable to those resulting from training programs that directly targeted text reading, confirming that accelerating the underlying cognitive processes shared by RAN and reading, such as quick lexical retrieval and automatized visual scanning, transfers efficiently to better reading skills.^6^^,^^24^^,^^25^ Additionally, action video game (AVG) training, known to improve attentional control, also resulted in subsequent gains in RAN performance, demonstrating that domain-general interventions targeting processing speed can causally enhance RAN and, consequently, reading fluency and accuracy in children with DD.^26^^,^^27^^,^^28^^,^^29^

Numerous case-control and correlational studies demonstrate that many individuals with DD exhibit deficits in motion perception tasks, such as coherent motion (CM) detection, compared with typically reading controls.^30^^,^^31^^,^^32^^,^^33^^,^^34^ These impairments point to a dysfunction in the magnocellular-dorsal (MD) pathway, a fast-conducting visual stream originating in large retinal ganglion cells that projects through the magnocellular layers of the lateral geniculate nucleus (LGN) to the primary visual cortex (V1) and the posterior parietal cortex (PPC).^35^^,^^36^ This specialized pathway is sensitive to low-contrast, high-temporal-frequency stimuli and is responsible for visuospatial attention, object localization, and the rapid timing of visual events required to control eye movements and stabilize binocular fixation during reading.^32^^,^^37^ In individuals with DD, there are deficits in this pathway, leading to visual perceptual instability, where letters may appear to move, blur, or overlap on the page.^32^

The robustness of this association is reinforced by converging psychophysical, anatomical, and neuroimaging evidence. Psychophysically, a significant proportion of readers with dyslexia show impaired processing of high-temporal, low-spatial-frequency stimuli, most reliably indexed by CM detection tasks that predominantly target the MD pathway.^31^^,^^32^^,^^38^ A meta-analysis by Benassi et al.^39^ reported a large mean effect size (d = 0.747) for CM deficits in dyslexia; however, rather than being a universal feature, this sensory impairment characterizes a specific subtype of continuum of the disorder, with prevalence estimates varying between approximately 16% and 75% of dyslexic individuals.^27^^,^^31^^,^^39^^,^^40^

Neurobiologically, postmortem analyses revealed that magnocellular neurons in the LGN of individuals with DD are fewer, smaller, and more disorganized, while parvocellular layers remain intact.^35^^,^^41^ Consistent with this anatomical evidence, functional magnetic resonance imaging (fMRI) studies have shown reduced activation of the LGN, V1, and motion-sensitive cortical area V5/middle temporal area (V5/MT) in response to moving stimuli in readers with dyslexia.^42^^,^^43^^,^^44^ Furthermore, reduced direct connectivity between left LGN and V5/MT has been observed in DD.^45^ Altered motion processing has also been observed in electroencephalogram (EEG) recordings of DD.^46^

Beyond binary case-control comparisons, correlational studies in unselected school populations comprising a representative continuum of children recruited regardless of their reading status have demonstrated graded relationships between motion perception and reading-related skills. These children’s CM detection thresholds were positively and non-linearly related to the likelihood of making orthographically inconsistent letter errors, even after controlling for age, IQ, reading level, and phonological awareness.^47^ These findings indicate that visual motion sensitivity covaries with reading-related performance across the population, rather than being an artifact of extreme group selection. However, correlational evidence alone cannot establish whether MD deficits are a cause or a consequence of reading failure.

To address this limitation, researchers have increasingly adopted longitudinal designs to determine whether MD pathway impairments precede reading acquisition. Studies of children at familial risk for dyslexia have shown reduced sensitivity to CM and frequency doubling (FD) stimuli before the onset of formal reading instruction.^30^^,^^38^^,^^48^ FD stimuli are thought to selectively engage low-level magnocellular M(y) retinal cells, providing an index of early visual encoding within the MD system.^49^ Critically, a prospective longitudinal study demonstrated that reduced CM sensitivity measured in kindergarten significantly predicted dyslexia diagnoses in third grade, whereas early literacy skills did not predict later motion sensitivity.^30^ Cross-lagged path analyses further confirmed this directionality: kindergarten CM sensitivity predicted first-grade letter knowledge but not vice versa.^30^

Importantly, the predictive strength of visual measures appears to depend on the level of processing assessed. Kevan et al.^38^ found that FD sensitivity—a lower-level magnocellular measure—predicted later reading accuracy and nonword reading, whereas CM performance did not. This suggests that early deficits in basic visual encoding may be particularly critical during the initial stages of reading acquisition, while higher-level motion integration may be more plastic and influenced by reading experience itself. Consistent with this interpretation, CM deficits observed in at-risk children often diminish following exposure to reading instruction, indicating that dorsal stream function can be refined through literacy experience despite its causal role.^30^

Finally, experimental intervention studies, the only designs capable of directly establishing causality, provide decisive evidence for a causal role of the MD pathway in reading development. Gori et al.^27^ demonstrated that visual motion perception in pre-readers predicted later reading outcomes independently of phonological skills. Crucially, they showed that targeted AVG training selectively enhanced MD function and produced concurrent improvements in reading performance in children with DD. This causal interpretation is further strengthened by evidence that MD deficits are present in pre-readers who later develop dyslexia but are absent in illiterate adults who have never learned to read, ruling out reduced reading experience as a sufficient explanation.^50^ However, this causal hypothesis remains debated, as other intervention studies suggest that motion processing deficits may be a consequence of impoverished reading experience or an independent correlate rather than a proximal cause.^51^^,^^52^

Our previous research suggests that reading direction influences visual perception, as neurotypical left-to-right readers show a right visual field (RVF) advantage for motion detection, a pattern not observed in individuals with dyslexia.^53^ While the link between motion sensitivity and reading ability is well established in adults, its role in early childhood remains debated. A key question is whether motion perception deficits are a cause or consequence of poor reading development.

Research on visual orienting demonstrates a tightly coupled relationship between covert attentional shifts and overt foveal eye movements, a relationship that is essential for understanding both reading acquisition and the difficulties observed in DD.^27^^,^^32^^,^^36^^,^^54^^,^^55^^,^^56^^,^^57^ Covert attention typically shifts to a spatial location first, enhancing processing even before the eyes move; overt foveation then follows the locus where covert attention has already been deployed.^57^^,^^58^^,^^59^^,^^60^ This covert orienting process is highly time-sensitive, with efficiency changes detectable within as little as 50 ms and peak orienting (maximum shift in attention efficiency; when the covert movement of attention provides the greatest relative benefit to the processing efficiency of a newly attended location) occurring around 150 ms after stimulus onset.^57^^,^^59^^,^^61^ These rapid shifts depend on a large-scale neural network centered in the PPC—a hub of visuo-spatial attention.^56^^,^^62^^,^^63^^,^^64^^,^^65^

In DD, a substantial body of evidence indicates that this visuo-spatial attention system is impaired, likely due to underlying dysfunction in the MD pathway. These impairments manifest as slower or less efficient attentional shifting, which in turn disrupts the rapid and precise selection of letters during reading.^27^^,^^56^^,^^66^^,^^67^ Critically, prospective longitudinal studies show that deficits in visuo-spatial attention precede formal reading instruction and significantly predict later DD diagnoses, indicating a causal role of MD pathway functioning in literacy development.^27^^,^^30^^,^^54^^,^^55^^,^^56^ Franceschini and Bertoni demonstrated a causal relationship between improvements in AVG and gains in reading skills during training in children with DD.^68^ Experimental intervention studies further strengthen this causal interpretation: AVG training, which enhances MD pathway efficiency and improves temporal attention, produces gains in reading fluency and phonological decoding.^26^^,^^27^^,^^29^^,^^69^^,^^70^ These benefits have been observed even in pre-readers at risk for dyslexia and were recently shown to be boosted by neuromodulation targeting the PPC in adults with DD.^71^^,^^72^ These findings underscore the PPC’s critical role as a controller of visuo-spatial attention underlying efficient reading networks.^54^^,^^72^^,^^73^^,^^74^

AVGs are uniquely defined by specific game mechanics, including high speed, fast-moving objects, spatial and temporal unpredictability, and a high degree of perceptual, cognitive, and motor load, often emphasizing peripheral processing and divided attention.^75^^,^^76^^,^^77^^,^^78^This specific genre places heavy demands on perceptual and attentional skills, serving as an ideal environment for enhancing spatial and temporal attention.^75^^,^^77^ These games promote learning by engaging reward systems and enhancing the efficiency of mechanisms like the accumulation of multisensory evidence or processing speed.^26^^,^^79^ Converging meta-analytic evidence confirms that habitual AVG players demonstrate enhanced cognitive skills, showing a large positive effect size (*g* = 0.64) compared with non-players in cross-sectional studies.^80^ Furthermore, intervention meta-analyses, focusing strictly on training contrasting AVGs with active control groups (like other commercially available non-action games) establish a causal link, showing that AVG training results in significant improvements in cognitive skills.^76^^,^^80^ These effects are most robustly observed in domains linked to the MD pathway, such as perception, spatial cognition, and top-down attention.^76^^,^^80^ Crucially, experimental intervention studies focused on DD provide evidence for causality by demonstrating that manipulating MD function directly impacts reading outcomes.^27^ For instance, targeted perceptual learning based on adaptive CM detection, designed to train the MD pathway, improved reading skills in adults with dyslexia.^27^ Similarly, AVG training, which enhances MD pathway function and attentional control, led to significant improvements in CM performance and subsequent gains in reading skills in children with DD.^27^ These intervention-induced visuo-attentional enhancements were found to account for a large portion (63%) of the variance in reading improvement in trained adults with dyslexia.^27^ A recent meta-analysis synthesizing nine intervention studies confirms these benefits in children with DD, showing that AVG training leads to a medium-to-large improvement in visual attention (VA, *g* = 0.72) compared with active control groups.^78^ Importantly, this meta-analysis also documented far-transfer effects, with significant improvements in RS (*g* = 0.44) and phonological processing (PP, *g* = 0.45) following AVG training.^78^ Most recently, AVG training proved effective as a preventive measure in pre-readers at risk for DD, resulting in significant enhancements and subsequent normalization of phonemic awareness compared to passive and active control groups.^71^ This finding suggests a causal effect of multisensory attentional training on core reading predictors early in development.^71^ These findings align with contemporary formulations of the magnocellular theory of DD, which posit that atypical development of MD pathway leads to impaired temporal processing and visuospatial attentional control, thereby disrupting the rapid selection of orthographic information required for fluent reading.^38^^,^^48^ Within this framework, AVG training may act by strengthening MD-driven attentional mechanisms, offering a mechanistic explanation for the observed improvements in both VA and reading-related outcomes.^26^^,^^27^^,^^70^^,^^76^

To clarify the relationship between motion perception, eye movement control, and reading readiness, we conducted an experiment in kindergarten children before formal reading instruction. Specifically, we assessed motion detection abilities and eye movement patterns during an AVG task and compared them to RAN performance. By this examination, we aim to determine whether motion perception and eye movement patterns—particularly the RVF advantage for motion detection—are already present in pre-readers. If these visual processing asymmetries emerge before formal reading instruction, this would provide further support for the hypothesis that motion perception causally influences reading skill development. Furthermore, identifying motion perception and eye movement deficits in pre-reading children could guide early interventions to mitigate future reading difficulties.

## Results

This study investigated reading-related cognitive processing in thirty-four kindergarten children who completed the RAN test and the AVG Fruit Ninja (FN) while their eye movements were measured.

### Action video gaming and rapid object naming are significantly correlated in children aged 4 to 6 years

**Figure 1:**
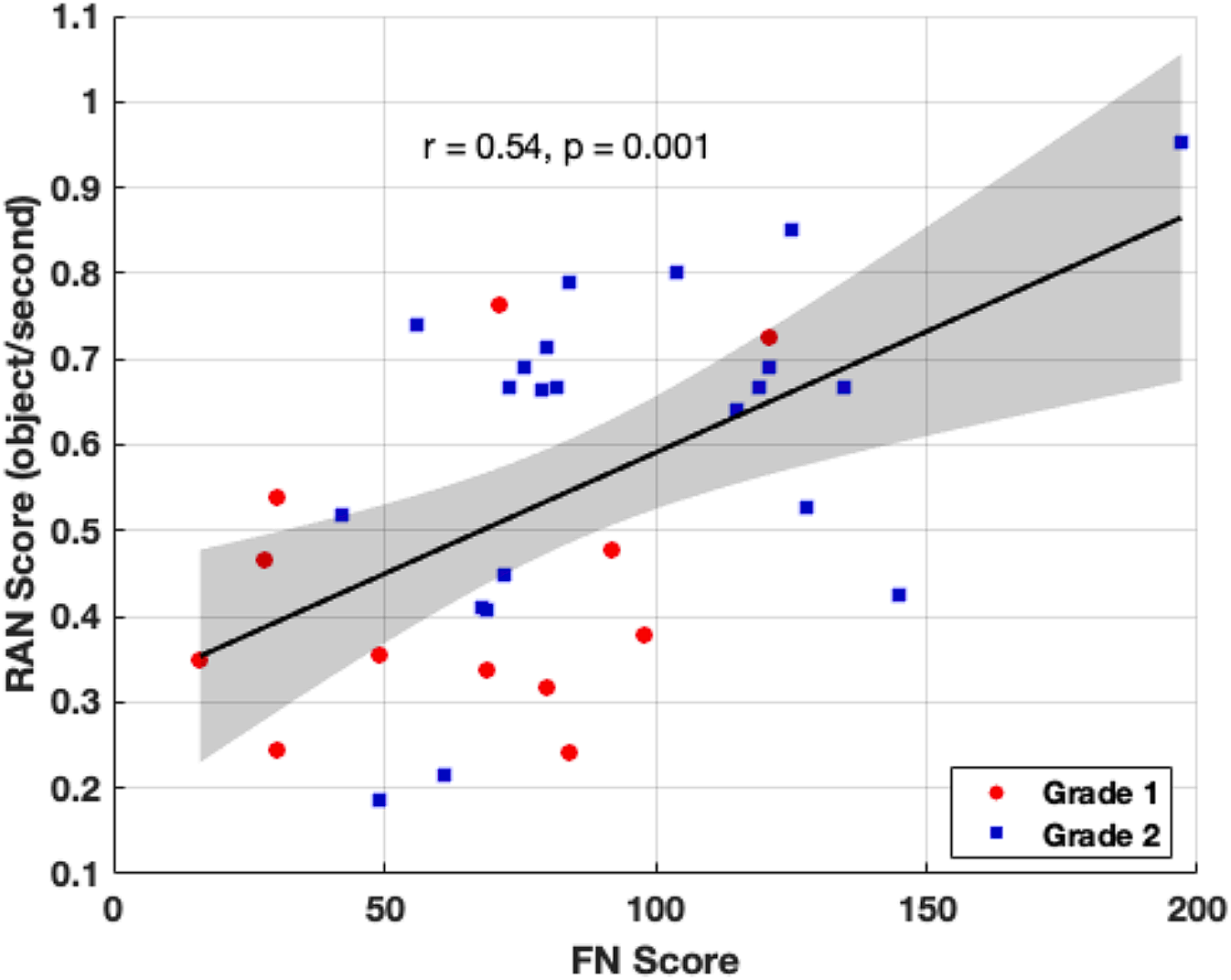
Performance during FN video gameplay predicts rapid naming during the RAN test Each red dot represents a single subject in grade 1 and each blue dot represents a single subject in grade 2. r and p values shown in the panel are the Pearson correlation coefficient and its associated two-tailed p value. Shaded areas indicate the 95% confidence intervals (CIs) of the linear regression fits.

Children who performed better on the RAN test also tended to achieve higher scores in the FN game during the 20-min sessions. A statistically significant positive correlation (r = 0.54, *p* = 0.001) confirmed that quicker object naming ability was associated with better performance in this fast-paced, visually dynamic video game. This relationship was consistent across the two assessed grade levels, as indicated by a regression analysis that showed the same trend regardless of educational stage (Figure 1). This first result therefore supports the view that cognitive processing during action video gaming and reading skills co-develop at kindergarten stage. Video game performance, as assessed with the FN score, is reliant on several distinct cognitive skills, we wanted to test more directly the hypothesis that motion detection skills are indeed a critical cause for succeeding at FN and performing well during RAN. To do so, we took advantage of eye movement data collected simultaneously during FN and RAN performance and applied machine learning algorithms (see STAR Methods) to identify the type of object (fruit or bomb), position in the visual field (central vs. peripheral and left vs. RVF).

### Object foveation as critical factor for succeeding in video game play and object naming

**Figure 2:**
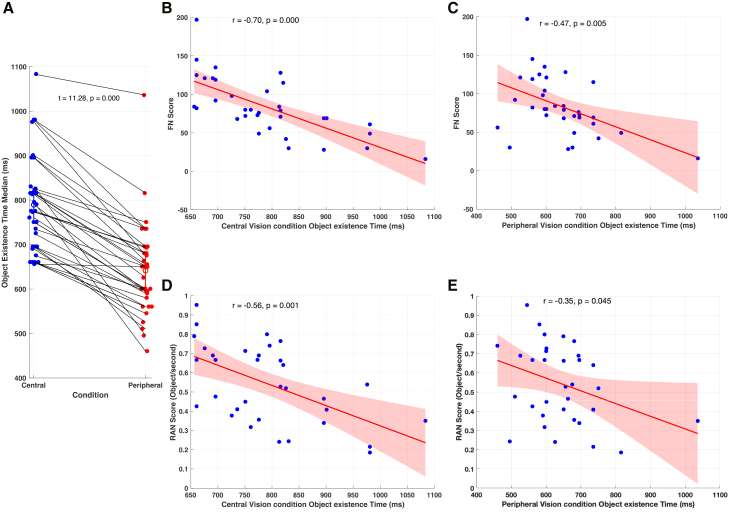
Comparison of central and peripheral vision conditions for object existence times and FN and RAN scores (A) Median object existence time (ms) for each subject comparing central versus peripheral conditions. The central condition refers to instances where the subject had fixated on the object, while the peripheral condition refers to instances where no fixation was made on the object. A significant difference is observed, with responses in the peripheral condition likely being faster than in the central condition, as indicated by shorter object existence times. t and p are the statistic and two-tailed p value from a paired t test. (B) Negative correlation between FN score and the median object existence time for objects on which the subject fixated before responding. r and p are the Pearson correlation coefficient and its associated two-tailed p value. (C) Negative correlation between FN score and the median object existence time when the subject responded without fixating on the object. (D) Negative correlation between RAN score and the median object existence time in the central condition. (E) Weaker negative correlation between RAN score and the median object existence time in the peripheral condition. Shaded areas indicate the 95% CIs of the linear regression fits.

A key aspect of FN gameplay consists of slicing moving fruits that appear at random intervals and locations on the screen. While central vision is optimized for accurate object identification, motion analysis is often better in peripheral vision.^81^^,^^82^ To clarify the role of central vs. peripheral vision during FN gameplay we examined the object existence time as a proxy for behavioral response times (see STAR Methods). Indeed, object existence times, and therefore, most likely behavioral responses were much faster when children relied on their peripheral vision, slicing fruits without directly fixating on them, compared with when they fixated centrally before responding (t(33) = 11.28, *p* < 0.001; Figure 2A).

However, during central fixation, object existence time was significantly correlated with FN score (r = –0.70, *p* < 0.001; Figure 2B). Under the peripheral vision condition, this correlation with FN score was moderate (r = –0.47, *p* = 0.005; Figure 2C). Similarly, during central fixation, object existence time was significantly correlated with RAN performance (r = –0.56, *p* = 0.001; Figure 2D), while the correlation was weaker under the peripheral condition (r = –0.35, *p* = 0.045; Figure 2E).

Therefore, faster response to the foveated objects during FN gameplay appears to be a critical factor not only for succeeding in FN gameplay, but also for predicting better object naming performance during RAN.

### Children had shorter saccadic reaction times to objects moving in their right visual field

**Figure 3:**
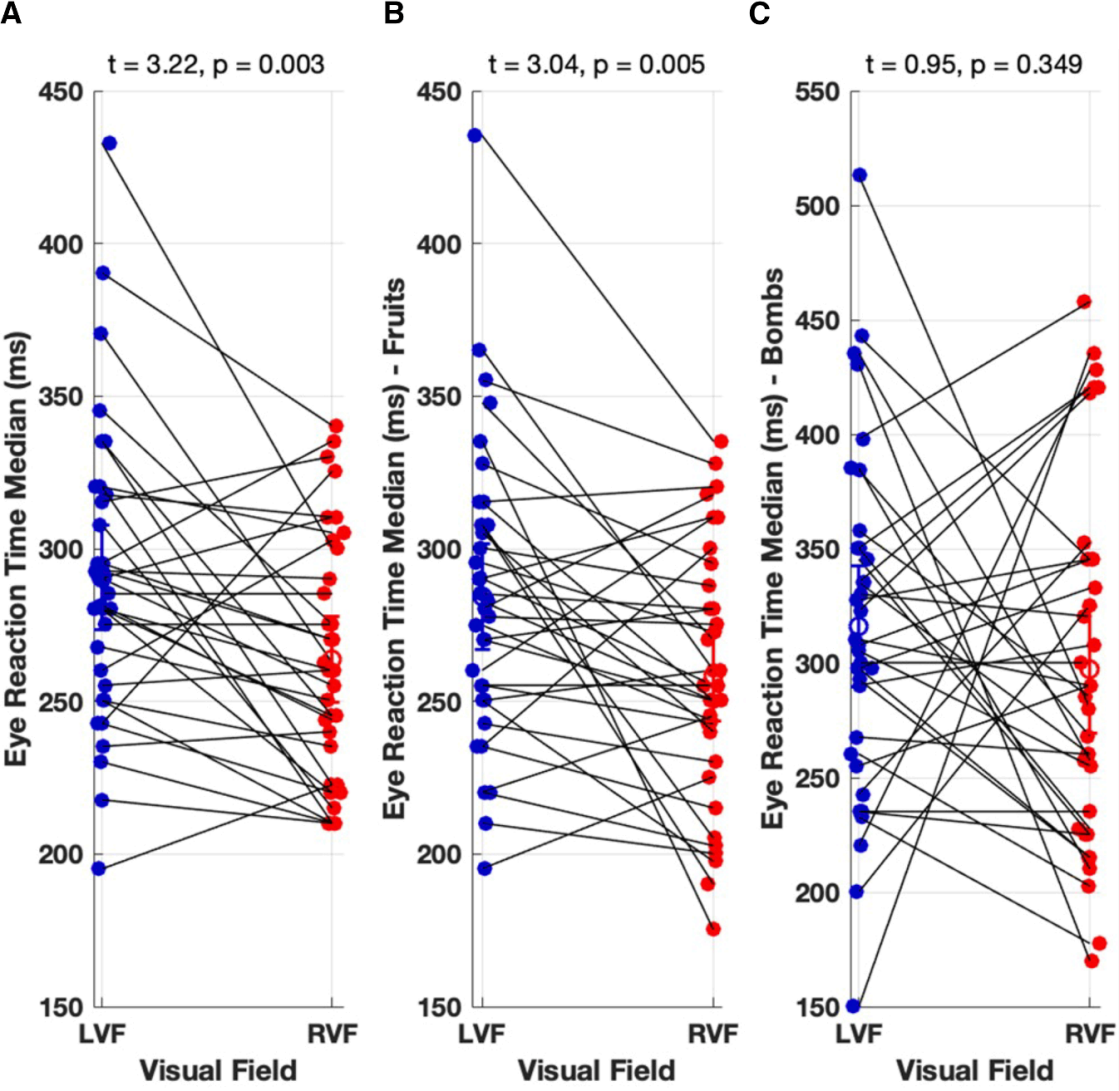
Visual field comparisons of saccadic eye reaction times (RTs) during action video gameplay (A) Median eye RT (ms) of each subject for both fruits and bombs combined. Overall saccadic RTs are shorter in the RVF. (B) Median eye RT (ms) of each subject specifically for fruits. A significant difference is observed, with lower eye RTs in the RVF compared with the LVF. (C) Median eye RT (ms) of each subject for bombs. No significant difference is observed between stimuli in the LVF and RVF. Each subplot shows the t statistic and *p* value from the paired *t* test. Error bars represent the 95% CIs of the mean. Each line connects the two conditions for a single subject; blue and red dots indicate LVF and RVF medians, respectively.

To understand the role of eye movement control in greater detail, we looked at the specific events occurring during FN gameplay. Saccadic eye movements can be crucial to identify the visual object as a fruit to be sliced or as a bomb, for which responses should be withheld. Any of these objects can occur either in the left visual field (LVF) or in the RVF. We, therefore, measured saccadic RT as the interval between when a stimulus appears in the visual field and the initiation of the first saccade that lands on it. Our analysis revealed clear differences between the LVF and the RVF, depending on the type of object. When considering all stimuli combined (fruits and bombs), children’s eyes moved significantly faster to objects in the RVF compared with those in the LVF (t(33) = 3.22, *p* = 0.003; Figure 3A), confirming our previous observation in adults using controlled motion stimuli.^53^ In the present sample, an RVF advantage was observed in 26 of the 34 tested children; among left-handed children, 3 of 5 showed the RVF advantage.

When focusing the analysis on fruits specifically, this RVF advantage remained more pronounced, with children showing quicker eye movements to fruits appearing in the RVF compared with the LVF (t(33) = 3.04, *p* = 0.005; Figure 3B). However, this difference was not significant for bombs, for which children were asked to withhold slicing in order to excel in the game (t(33) = 0.95, *p* = 0.394; Figure 3C).

## Discussion

Our study provides correlational evidence for the role of motion perception and eye movement control in early reading development. Consistent with Gori et al.,^27^ we found that better performance in the RAN task was significantly associated with higher scores in an AVG, reinforcing the idea that motion perception plays a foundational role in reading readiness. Importantly, our study extends these findings by demonstrating that object foveation—the act of directly fixating on an object before responding—is a critical factor for both video game success and rapid object naming. Children who had faster hand response times under the condition that they fixated on moving objects before responding, also exhibited superior RAN performance. The relationship with RAN performance was weaker when participants relied on peripheral vision during FN gameplay.

A central component of FN gameplay is the necessity to slice rapidly appearing fruits across the visual field. Because object identification relies on central (foveal) vision whereas motion sensitivity is often superior in peripheral vision,^81^^,^^82^ FN performance depends on the interplay between peripheral motion monitoring and timely overt foveation. Our analysis of object existence time—a proxy for behavioral response speed—showed that children responded significantly faster when slicing fruits without directly fixating on them, i.e., relying on peripheral vision. However, the key finding is that only when children foveated the fruits did object existence time correlate with overall FN performance and with RAN naming speed. A weaker relationship was observed under peripheral-only responding, likely because participants performed more efficiently using peripheral vision, whereas the central-vision condition better captured between-subject variability in performance.

These results indicate that foveation, not peripheral responding alone, emerges as an important mechanism linking VA, FN performance, and object naming efficiency. This aligns directly with the visual orienting model: effective task performance requires a precise covert-to-overt orienting sequence, in which covert attention selects the target and the eyes then move for high-resolution processing.^57^ Children who demonstrate more efficient foveation in FN—not just fast reactions—also show better RAN performance, suggesting that shared visuo-attentional mechanisms support both rapid object naming and efficient action-based responses. Within the context of DD, where sluggish or inefficient attentional shifting is well documented, these findings reinforce the view that impairments in the MD-PPC attentional system disrupt the speed and coordination of covert and overt orienting,^27^^,^^32^^,^^83^ leading to downstream difficulties in reading and rapid naming.^55^^,^^69^

Additionally, we observed an RVF advantage in saccadic RTs, with children responding more quickly to objects appearing in their RVF compared with the LVF. This asymmetry, which was particularly pronounced for fruit stimuli, aligns with prior research showing that motion perception and visual processing efficiency are lateralized in the brain.^53^ Notably, this RVF advantage did not extend to bomb stimuli, for which children were required to inhibit responses, further suggesting that the observed asymmetry reflects active target detection rather than general attentional bias.

The left hemisphere (LH) demonstrates clear specialization for linguistic functions, including speech and the serial processing of stimuli, which are fundamental to fluent reading.^32^^,^^84^^,^^85^ Conversely, the right hemisphere (RH) is generally dominant in the large-scale visuospatial attention network and global processing, including functions of the MD pathway responsible for motion perception.^32^^,^^35^^,^^59^^,^^64^^,^^86^ The observed advantage for oculomotor behavior in the RVF, presumably mediated by the LH, during visual tasks may initially appear counterintuitive.^87^ However, supposing this RVF/LH advantage is linked to the learned demands of serial oculomotor control required for reading in left-to-right scripts, which requires the rapid selection of letters by directing attention and subsequent foveation predominantly into the RVF.^87^^,^^88^^,^^89^ Reading then may act as a causal mechanism, shaping the attention network to optimize the sequential shifting of covert attention—a process that precedes the overt eye movement—to prepare for foveating the next word or letter in sequence.^57^^,^^58^^,^^60^^,^^87^ The dorsal stream, which feeds the PPC and controls the planning of these orienting movements, is thus mobilized and leveraged by the LH’s specialized requirement for rapid, serial visuo-verbal integration during literacy acquisition.^32^^,^^62^^,^^87^^,^^90^ This acquisition tunes the dorsal stream to act as a serial attentional gate, modulating visual signals as early as the V1 to prevent the ventral stream from being confounded by simultaneous information arriving from across the entire visual field.^83^^,^^91^ Such tuning transforms naturally “random and anarchic” visual search into a systematic sequential process,^91^^,^^92^ resulting in faster and more significant engagement of left-hemisphere speech-motor areas for RVF inputs compared with the LVF.^93^

The efficiency of this system is directly correlated with reading proficiency; for example, skilled readers show lateralization of temporal processing favoring the RVF,^53^ while individuals with dyslexia, who often exhibit pre-existing structural and functional deficits in the MD pathway and abnormal brain lateralization,^27^^,^^35^^,^^43^^,^^45^^,^^94^^,^^95^^,^^96^ typically show a lack of this RVF perceptual asymmetry.^53^^,^^88^ Specifically, Müller-Axt et al., have identified reduced structural connectivity in the direct pathway linking the left LGN to the left V5/MT, while connections to the V1 remain intact; notably, the strength of this specific left V5/MT-LGN connectivity correlates with RAN abilities.^45^ This pattern indicates that the RVF/LH advantage is a product of reading acquisition leveraging the LH’s serial processing capabilities and the MD pathway’s oculomotor control functions, rather than a generalized intrinsic bias for motion processing in the LH.^87^ However, dyslexia is frequently associated with pre-reading deficits in the MD pathway, which is critical for motion processing and attentional control.^27^^,^^30^^,^^38^ The defective connectivity of the MD pathway, specifically reduced structural connectivity from the left LGN to motion area MT/V5, suggests a crucial role in the acquisition of reading skills.^35^^,^^45^^,^^94^ These structural and functional abnormalities, which often show an LH bias, may hinder the normal development of temporal perceptual asymmetry, resulting in uniformly poor temporal processing across both hemifields in dyslexic individuals.^43^^,^^53^

Existing cross-linguistic evidence confirms that reading direction does dramatically shape visual field asymmetries: bilingual readers of both left-to-right (e.g., English) and right-to-left (e.g., Farsi, Arabic, and Hebrew) scripts often exhibit no visual field asymmetries, while right-to-left readers show a mirrored advantage in the LVF.^53^^,^^87^^,^^97^ Moreover, languages with visually complex scripts, such as Arabic, may pose additional challenges to reading acquisition and automatization, resulting in the RH being less involved in word identification compared with simpler orthographies, leading to slower reading speeds despite possible phonological advantages.^98^ However, the presence of the RVF advantage in our preliterate sample, before the German orthography (a transparent system) could have exerted a more significant shaping influence, supports the view that the underlying neural network for visuospatial attention (PPC and the MD pathway) is pre-tuned for efficient processing of stimuli in the reading direction, potentially indicating a neurobiological precursor to literacy success, rather than a mere cultural artifact.^53^^,^^72^^,^^99^

In the present study, the term “cognitive functions” has been used to refer to domain-general abilities such as attention, working memory (WM), processing speed, inhibition, and motor planning that are commonly assessed at the behavioral level. The term “mechanism” is used to denote the dynamic processes through which these functions are implemented during task performance, including serial attentional shifting, visuomotor coordination, evidence accumulation, and the temporal coupling of eye movements with decision and motor execution. The efficiency of the RAN task, a more significant longitudinal predictor of early literacy, is heavily reliant on the seamless coordination of several complex, domain-general cognitive skills, including attention, WM, processing speed, and motor planning.^5^^,^^6^^,^^100^^,^^101^^,^^102^ RAN is considered a “microcosm of reading” because it necessitates rapid multimodal integration, visuo-verbal processing, and the coordination of processes such as attentional shifts, lexical access, saccadic movements, and articulatory planning.^6^^,^^14^ The need for serial processing during RAN imposes continuous demands for inhibition—to suppress naming the previous item—and rapid disengagement of attention, followed by its deployment to the next stimulus.^101^^,^^103^ Moreover, RAN explicitly relies on rapid decision-making and action planning required for time-constrained lexical retrieval and motor output, a factor particularly relevant for fluency.^101^^,^^104^^,^^105^ While our research focused on observable phenomena like oculomotor behavior and reaction time (RT) during a visual motion task (such as FN-style game, which involves high-speed, unpredictable movement, and high perceptual/motor load), these underlying unmeasured skills are precisely the domain-general factors implicated in the “common cause” models of the RAN-reading relationship.^101^^,^^106^ These models posit that factors such as processing speed, WM, and attention act as distal variables that predict both RAN performance and reading fluency, often by mediating the influence of RAN on linguistic skills like phonological and orthographic processing.^100^^,^^101^ Alternatively, the visual-attentional span (VAS) theory suggests that reading fluency impairments arise from a deficit in simultaneously identifying multiple letters within the visual field,^99^ and that attention-intensive activities such as AVGs may enhance reading by improving VAS performance and the parallel processing of visual information crucial for foveation.^28^^,^^99^^,^^107^ Our study’s key finding that better RAN performance correlated significantly with superior AVG performance provides direct empirical justification for these “common cause” models,^26^^,^^78^^,^^101^^,^^105^^,^^106^ confirming that the rapid visuo-verbal integration central to RAN shares underlying domain-general mechanisms, such as processing speed,^100^^,^^101^ attentional control,^75^^,^^101^^,^^105^ and the “accumulation of evidence,”^28^^,^^72^^,^^79^ with complex, dynamic visual motor tasks.^75^^,^^78^^,^^99^ Furthermore, the emergent finding that pre-reading children exhibited faster RTs to the RVF is critical, as the RVF advantage reflects the LH specialization for temporal perceptual resolution,^108^^,^^109^^,^^110^^,^^111^^,^^112^^,^^113^ which is necessary for the serial visual scanning and rapid foveation characteristic of left-to-right reading.^53^^,^^87^^,^^89^^,^^105^ This early asymmetry suggests that the visual-attentional system is already tuning for the efficiency requirements of literacy, linking oculomotor dynamics and rapid motion perception in the preferred reading direction directly to RAN proficiency, and thus validating the study’s focus on visual-attentional factors as proximal determinants of reading readiness.^30^^,^^87^^,^^99^

Our study involved pre-reading kindergarten children (aged 4–6 years) with minimal or no formal reading instruction, evidenced by their status as pre-readers who, in similar longitudinal studies, knew on average fewer than three letters.^30^ The early existence of the RVF advantage in these preliterate German-speaking children, specifically for processing dynamic visual events is, therefore, unlikely to be a consequence of culturally ingrained left-to-right reading habits or extensive reading practice. Instead, this finding suggests that the neural specialization for rapid temporal processing in the LH, necessary for reading, may be developmentally emerging before reading acquisition fully solidifies the process, potentially reflecting an inherent specialization or pre-reading maturational milestone. Longitudinal studies examining MD deficits, which are linked to temporal processing and VA, confirm that impaired MD function is predictive of later reading difficulty before children learn to read, strengthening the claim for a causal, neurobiological determinant rather than an exclusively experiential consequence.^27^^,^^30^^,^^38^^,^^50^

Together, these findings suggest that early inefficiencies in motion perception and lateralized processing could serve as early indicators of later reading impairments.

### Limitations of the study

While our findings provide meaningful insights into the relationship between motion perception and reading readiness, several limitations must be considered. First, our sample size was relatively small, which limits the generalizability of our results. Larger-scale studies are needed to confirm these findings across more diverse populations. Second, 32% of children in our sample were bilingual (proficiency in French and German), which introduces potential variability in language development that might interact with visual processing and RAN performance. Future studies should consider monolingual and bilingual groups separately and directly compare their performance to clarify these effects. Third, while we aimed to assess motion perception as a core cognitive mechanism, we cannot fully rule out contributions from general intelligence or broader perceptual deficits that might influence both video game performance and RAN. Future research should, therefore, include standardized neuropsychological measures of nonverbal intelligence, visuospatial attention, WM, and processing speed to more precisely isolate visual motion-specific contributions from domain-general cognitive factors. The influence of sex on the study results was not systematically assessed due to the small sample size, which represents a limitation to the generalizability of the findings. Furthermore, the object detection algorithm used to track fruits and bombs was unable to directly capture the slicing action, relying instead on the duration of object existence as a representative for response time.

These insights highlight the importance of early interventions targeting motion perception and visual field asymmetries to support reading acquisition. Given that our study identified motion perception differences in pre-readers, this suggests that training programs, such as action video games aimed at strengthening motion perception may be particularly effective if implemented early. Gori et al.,^27^ Franceschini et al.,^26^ and Peters et al.^29^ provide further support for this approach, demonstrating that video game-based motion training can enhance reading skills in both pre-readers and older children with dyslexia. Future research should explore how such interventions influence long-term reading outcomes and whether targeted training can help restore typical hemispheric lateralization in at-risk children.

## Resource availability

### Lead contact

Further information and requests for resources and reagents should be directed to and will be fulfilled by the lead contact, Michael Christoph Schmid (michael.schmid@unifr.ch).

### Materials availability

This study did not generate new materials.

### Data and code availability

• The dataset analyzed in this study is available upon request from the lead contact.
• The custom object detection and tracking code used in this study is deposited at Zenodo (https://doi.org/10.5281/zenodo.20758595) and is publicly available as of the date of publication.
• Any additional information required to reanalyze the data reported in this paper is available from the lead contact upon request.

## Acknowledgments

We acknowledge Roboflow, Inc. and the creator of the Merged-FruitNinja dataset for providing access to the dataset used in this research via the Roboflow Universe platform. The dataset was used under the Creative Commons Attribution 4.0 (CC BY 4.0) license, which permits both non-commercial and commercial use with appropriate attribution. We thank Antonina Kaplan, Lisa Luethi, Julia Kolly, Aline Sophie Zimmermann, and Salome Hausheer for their assistance in data collection. We thank Olivia Lecomte for her assistance in fine-tuning the object detection algorithm. We thank the children, parents, and teachers of Jura Schule Fribourg for their support of this project; our colleagues Drs. Nicolas Ruffieux and Julia Winkes for helpful discussions; and Fribourg’s Amt für deutschsprachigen obligatorischen Unterricht and the Service for Logopädie, Psychologie und Psychomotorik for support. This work was supported by a Swiss Government Excellence Scholarship awarded to N.C. and 10.13039/501100001711SNSF grant nos. 31NE30_203974 (ERA-net) and 310030_204544 to M.C.S.

## Author contributions

N.C. and M.C.S. drafted the manuscript. N.C. conducted the analysis, created all the figures, and presented the findings. S.R. and M.C.S. co-supervised the project. All authors contributed to the interpretation of results, provided insights, and reviewed the manuscript throughout its preparation.

## Declaration of interests

The authors declare no conflicts of interest associated with this article.

## Declaration of generative AI and AI-assisted technologies in the writing process

During the preparation of this work, the authors used ChatGPT in order to improve readability and language and BioRender to create the graphical abstract. After using this tool or service, the authors reviewed and edited the content as needed and take full responsibility for the content of the publication.

## STAR★Methods

### Key resources table

**Table:** 

REAGENT or RESOURCE	SOURCE	IDENTIFIER
**Deposited data**		

Object detection and tracking code (this paper)	This paper	https://doi.org/10.5281/zenodo.20758595
Roboflow FruitNinja training dataset	Roboflow Universe	https://universe.roboflow.com/yolo-j3jjb/merged-fruitninja/dataset/4; CC BY 4.0

**Software and algorithms**		

MATLAB R2022b	MathWorks	https://www.mathworks.com; RRID: SCR_001622
Python 3.9.20	Python Software Foundation	https://www.python.org; RRID: SCR_008394
YOLOv8s (Ultralytics v8.3.37)	Ultralytics	https://github.com/ultralytics/ultralytics
PyTorch 2.5.1	PyTorch	https://pytorch.org; RRID: SCR_018120
OpenCV 4.10.0.84	OpenCV	https://opencv.org; RRID: SCR_015526
Pandas	NumFOCUS	https://pandas.pydata.org; RRID: SCR_018214
Pupil Cloud	Pupil Labs	https://pupil-labs.com

**Other**		

Pupil Labs Neon eye-tracking glasses	Pupil Labs	https://pupil-labs.com/products/neon
iPad Pro 12.9-inch	Apple	N/A
Dyslexia Adult Screening Test (DAST); object naming subtest	Pearson Clinical Assessment; Nicolson and Fawcett^114^	ISBN-13: 978-0749113216

### Experimental model and study participant details

#### Human participants

This cross-sectional study was conducted by the Department of Neuroscience, Faculty of Medicine, University of Fribourg, after receiving ethics approval from the Business Administration System for Ethics Committees (BASEC), University of Fribourg, Switzerland (2023-00226). The study involved German-speaking children in the city of Fribourg, Switzerland, and investigated their developmental processes using eye-tracker recordings during gameplay performance and RAN performance. A total of 34 participants were included in the study, consisting of 18 females (53%) and 16 males (47%). The mean age of the participants was 5.29 years (SD = 0.71), with ages ranging from 4 to 6 years. Hand dominance was right-handed in 82% of participants (*n* = 28), left-handed in 15% (*n* = 5), and bilateral in 3% (*n* = 1). Thirty-five percent of the participants were first graders, and 65% were second graders. Regarding language background, the majority of participants reported German as their mother tongue (68%), with the rest reporting bilingual proficiency in French and German (32%). Family history of dyslexia was self-reported, with 76% of participants having no history, 12% reported a positive family history of dyslexia, and 12% were unsure (Table S1).

Participants were recruited through direct connections with schools. Parents provided written informed consent, and children gave verbal permission to participate. Children were included if they had normal IQ, normal or corrected visual acuity (e.g., with glasses: one subject), no neurological or psychiatric disorders, no sensory visual impairments, were not using medications, and were willing to cooperate. Eligibility was determined based on school records and teacher evaluations. Mobile Pupil Labs Neon eye tracking glasses (Pupil Labs, Berlin, Germany) were utilized throughout the entire session. The glasses were connected to an Android phone equipped with the Pupil Cloud Companion App, which collected data via a USB-C connection. Offset correction and brightness adjustment were performed before starting the experiment to achieve 1.3° accuracy. The device provided binocular eye video recordings at 200 Hz (concatenated in a single video stream of 384 × 192 px resolution) and scene interaction recordings at 30 Hz (1600 × 1200 px resolution with a field of view (FOV) of 103°). Gaze data were output in pixel space of the scene camera image, with the origin located in the top-left corner of the image. A 9-degrees-of-freedom IMU was integrated into the module and sampled at 110 Hz. It captured the inertia of the glasses, including translational acceleration, rotational speed, magnetic orientation, pitch, yaw, and roll. Based on the given scene camera’s horizontal and vertical FOV and resolution, the angular displacement for each pixel is approximately 0.064° both horizontally and vertically, relative to the scene camera’s FOV:

$$Horizontal\;size\;per\;pixel\;\left({}^{\circ}\right)=\frac{Horizontal\;FOV\;\left({}^{\circ}\right)}{Horizontal\;resolution\;\left(px\right)}=\frac{103}{1600}$$

Horizontalsizeperpixel(°)=0.064375°

$$Vertical\;size\;per\;pixel\;\left({}^{\circ}\right)=\frac{Vertical\;FOV\;\left({}^{\circ}\right)}{Vertical\;resolution\;\left(px\right)}=\frac{77}{1200}$$

Verticalsizeperpixel(°)=0.064167°

This study employed the object naming subtest from the Dyslexia Adult Screening Test (DAST),^114^ administrated on an iPad Pro 12.9-inch (resolution: 2048 × 2732 px, refresh rate: 120Hz). The test involved naming objects displayed on the screen, and it was conducted in German, as all participants were fluent in the language. The RAN task for each participant included an explanation, a preliminary trial to verify task comprehension, and the actual task performance, taking approximately 10 min within the overall session.

Participants were instructed to name pictures of objects as quickly and accurately as possible. The raw scores represented the time in seconds required to name all the objects, with a penalty of 5 s added for each error. The final score represented the number of objects named correctly per second, accounting for the errors made during the task.

Participants also engaged with ‘Fruit Ninja’ (FN) games for a continuous period of 20 min. In the study conducted by,^29^ the mobile application FN was employed as a research tool to examine its potential benefits on the reading abilities of children diagnosed with dyslexia. This application involves the task of slicing fruits that appear on the screen in a rapid and unpredictable manner, both temporally and spatially. The game’s mechanics not only require players to accurately slice fruits to gain points but also to dynamically track motion trajectories, switch their attention between specific targets and the broader game environment, plan and inhibit actions to avoid touching bombs, and make quick decisions to maximize their score.

All children have been given a gift at the end of the session, as a token of appreciation for their participation.

### Method details

#### Eye tracking methodology: Fixation detection algorithm and saccades main sequence for rapid automatized naming and FN

Fixations and saccades were automatically calculated in Pupil Cloud using an algorithm tailored for head-mounted eye trackers (see Algorithm description - 3.6.24 for details). The algorithm compensates for head movements, including those caused by the vestibulo-ocular reflex, ensuring robustness. Saccades were identified as gaps between fixations in the data. The relationship between saccade amplitude and peak velocity was analyzed during the RAN test and FN gameplay, with individual and aggregated main sequence plots color-coded by the ratio of average to peak velocity (Figure S1). Individual (Figures S1A and S1C) and aggregated (Figures S1B and S1D) plots illustrate saccadic behavior across tasks.

#### Object detection and tracking methodology

To analyze eye-tracking scene videos from FN gameplay, an object detection model was trained using YOLOv8s (Ultralytics, version 8.3.37) in Python 3.9.20, with PyTorch 2.5.1+cu124 and CUDA 12.4 for GPU acceleration.

The training dataset comprised 8,050 labeled images in COCO format, including 6,123 labeled images obtained from Roboflow Universe Merged-FruitNinja Dataset under the Creative Commons Attribution 4.0 (CC BY 4.0) license and 1,927 custom-labeled images. Of these, 6,497 images were used for training, and 1,553 for validation. Proper attribution was provided in compliance with Roboflow’s terms of use.

The model training was conducted on an NVIDIA GeForce RTX 3070 Ti GPU with a batch size of 4, an input resolution of 640 × 640 pixels, and 100 epochs. Data augmentations such as mosaic and random erasing were applied.

The trained YOLOv8 model was used to detect and track objects in FN gameplay videos captured from the scene camera of the Pupil Neon Eye-Tracking System. Videos were processed frame-by-frame using OpenCV (version 4.10.0.84), and object tracking was performed using the YOLO track method, which maintained object IDs across frames. Each detected object in every frame was represented by its bounding box, which included the center coordinates (x, y) and dimensions (width, height).

The object-tracking results were augmented with world timestamps provided by Pupil Cloud to synchronize with eye-tracking data. Using the pandas library in Python, event names from Pupil Cloud’s events.csv file were added to the object-tracking data based on timestamp matches. The events.csv file, provided by Pupil Cloud, was generated based on the start and end of recordings and the manually marked onsets of the RAN test and each FN game round.

Additionally, MATLAB R2022b was used to annotate eye-tracking data (gaze.csv, fixations.csv, and saccades.csv) with event names derived from events.csv. It was also employed for the subsequent analyses discussed in the following sections, as well as for generating all the plots.

#### Object detection model validation metrics

The trained YOLOv8 model achieved a mean Average Precision (mAP) of 99.1% at an Intersection over Union (IoU) threshold of 0.5, with precision and recall exceeding 98% for “Bombs” and “Fruits,” indicating its reliability and accuracy for object tracking during FN gameplay.

#### Object appearance, disappearance, and eye movement analysis

The onsets of appearance and disappearance for each object (fruits and bombs) were identified. For fruits, the disappearance time was defined as either when the subject successfully cut the fruit or when the fruit disappeared from the screen due to inaction. Eye coordinates at the time of each object’s appearance on the screen were analyzed relative to the object’s bounding box. Objects were categorized into the RVF or LVF based on their position relative to the eye coordinates at the onset of appearance. An object was classified as RVF if its center x-coordinate exceeded the eye’s x-coordinate, and as LVF if its center x-coordinate was smaller.

Based on oculomotor behavior, each object was classified into one of two conditions.

1. **Central Condition**: The participant made one or more fixations on the object at any point during the object’s presence.
2. **Peripheral Condition**: No fixation was made on the object throughout the entire duration of its existence.

Fixations on the object were determined by the overlap between the eye gaze coordinates and the object’s position. Figure S2 presents examples of the x-coordinates for both eye gaze and the object’s center. Figure S2A illustrates an RVF object with one saccade landing on it, and its eye-object x-coordinate correlation is shown in Figure S2B. Figure S2C depicts an LVF object with two saccades landing on it, with the corresponding correlation shown in Figure S2D.

#### Assessment of object existence time, eye reaction time, and their statistical analysis

To evaluate children’s interaction with objects during the FN task, existence time of the objects was calculated by measuring the duration between an object’s appearance and disappearance, regardless of whether it was cut or missed due to delayed response. This measure was used as an index of attentional processing efficiency, reflecting the time required to detect and respond to task-relevant stimuli under dynamic visual conditions. Importantly, object existence time captures attentional processing occurring both with and without overt eye movements, depending on the viewing condition.

The analysis focused on examining the relationship between RAN scores with FN performance, including FN scores and Existence times under both central and peripheral conditions during the FN task. Additionally, eye reaction time (RT) was assessed for objects in the central condition as a measure of overt attentional orienting and oculomotor initiation, by measuring the interval between object appearance and the onset of the first saccade toward it.

In the peripheral condition, object existence time primarily reflects covert attentional allocation without foveation.

RTs were measured in milliseconds, and objects with eye RTs below 100 ms or above 1000 ms were excluded from further analysis. Statistical analysis was conducted using Pearson’s correlation and paired t-tests to examine the relationship between these variables.

### Quantification and statistical analysis

All statistical analyses were performed using MATLAB R2022b. Object detection and tracking were implemented in Python 3.9.20 using YOLOv8s (Ultralytics, version 8.3.37). The sample size was *n* = 34 participants throughout all analyses, representing individual children.

Pearson’s correlation coefficients were used to assess relationships between FN gameplay scores, RAN performance, and median object existence times under central and peripheral vision conditions. Paired t-tests were used to compare median object existence times between central and peripheral conditions, and to assess visual field asymmetries in saccadic reaction times between the LVF and RVF. Statistical significance was set at *p* < 0.05. Effect sizes are reported as Pearson’s r for correlations. Shaded areas in scatterplots represent 95% confidence intervals of the linear regression fits. Error bars in boxplots represent 95% confidence intervals of the mean. All of the statistical details are reported in the results section and in the corresponding figure legends.
