# Supplementary material for: Motion perception and eye movements predict reading readiness: Evidence from pre-readers in an action video game task
Source: iScience. 2026 Aug 6;29(8):117063. doi: 10.1016/j.isci.2026.117063 (PMC13475668; doi:10.1016/j.isci.2026.117063)
Supplement: Supplementary file 1 [file mmc1.pdf]

iScience, Volume *29*

Supplemental information

Motion perception and eye movements predict reading readiness: Evidence from pre-readers in an action video game task

Niloufar Chamani, Samy Rima, and Michael Christoph Schmid

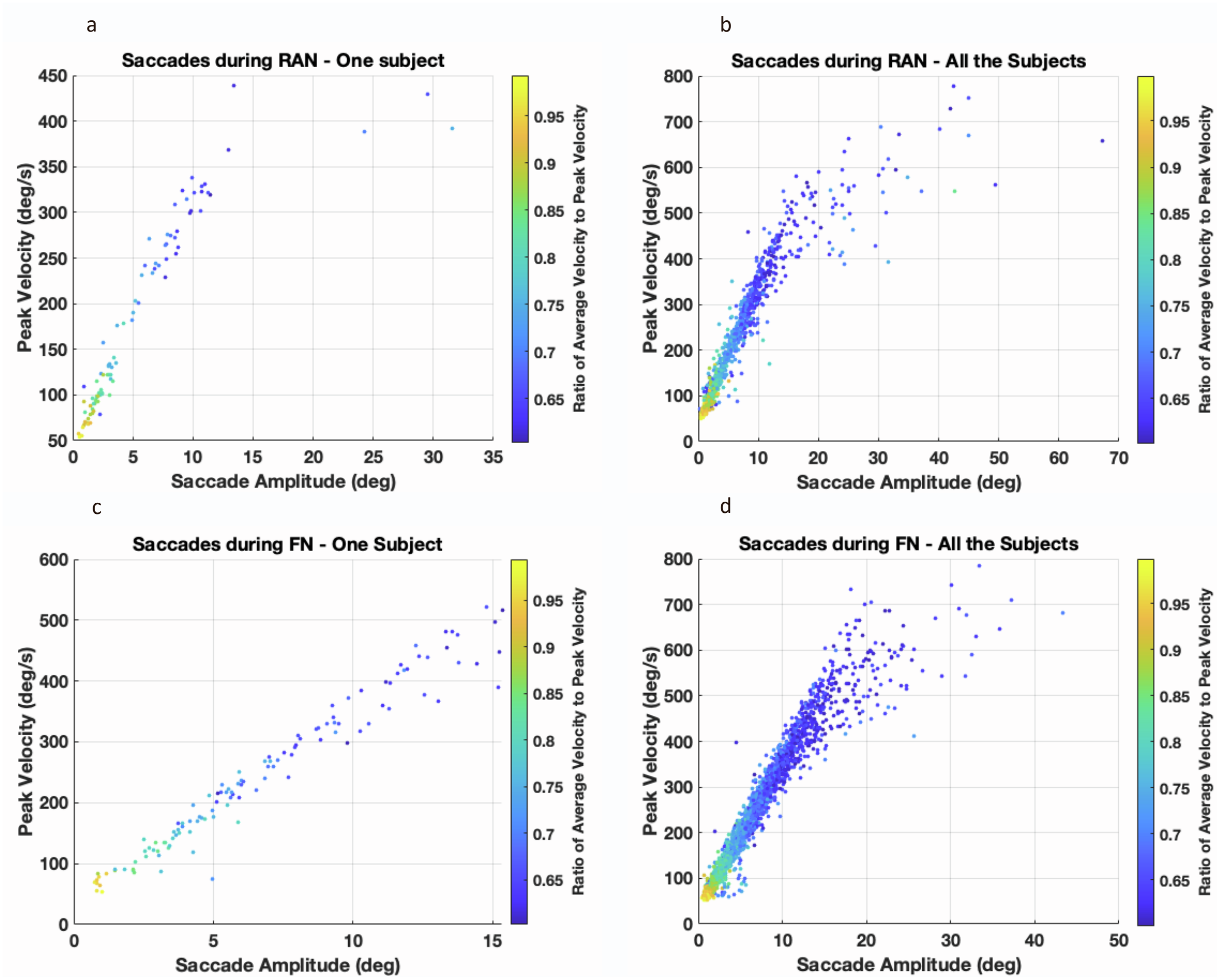

Figure S1. Main sequence plots illustrating the relationship between saccade amplitude (degrees) and peak velocity (degrees/second). (a) Main sequence for a single subject during the RAN test. **(b)** Aggregated main sequence for all participants during the RAN test. **(c)** Main sequence for one subject during FN gameplay. **(d)** Aggregated main sequence for all participants during the FN gameplay. Data points are color-coded based on the ratio of average velocity to peak velocity.

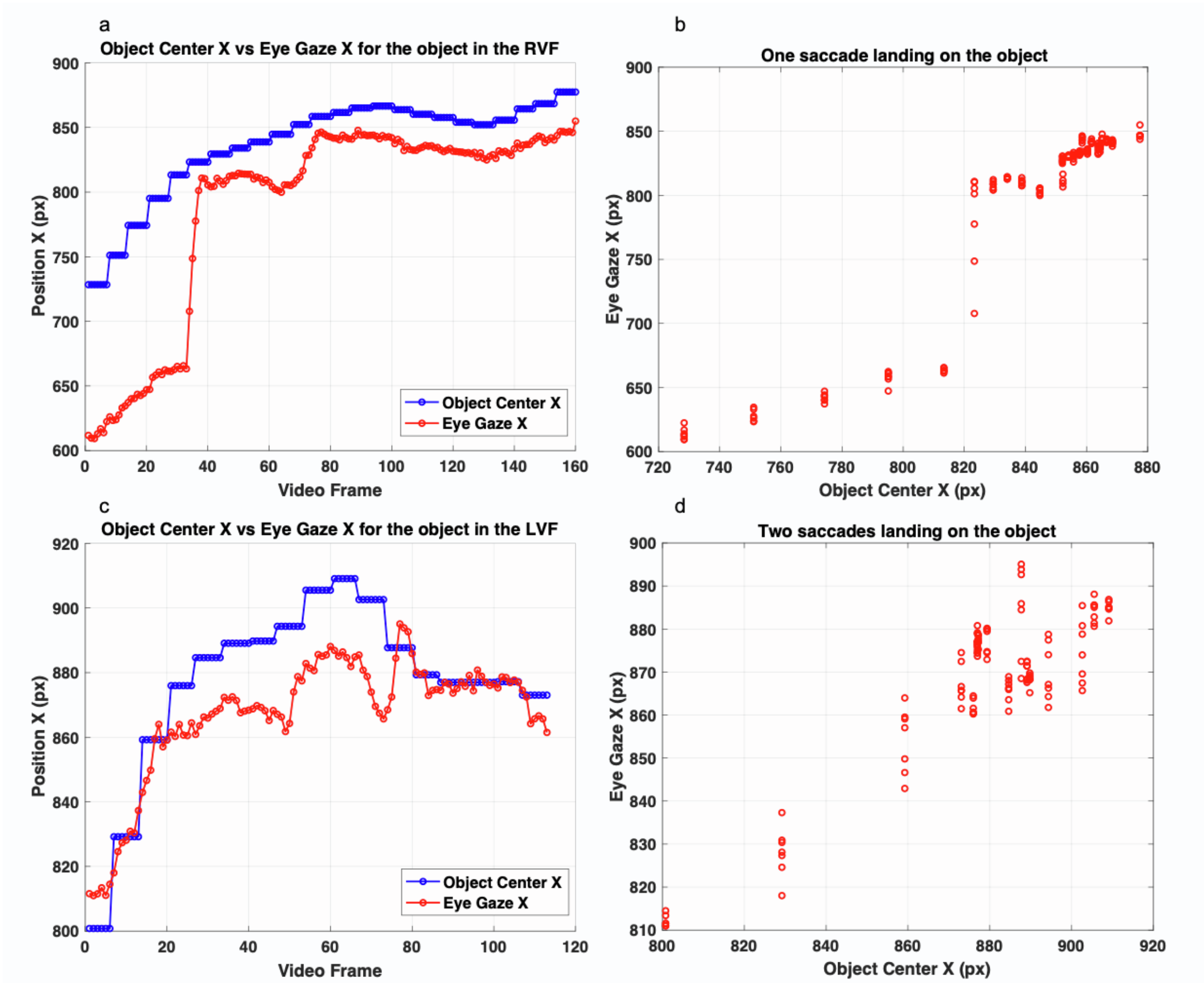

Figure S2. Examples illustrating the appearance and disappearance of objects on the screen, their relative positions to the eye's x-coordinate (left panels), and their corresponding eye-object x-coordinate correlations (right panels). (a) An object appeared in the right visual field (RVF), with the subject making one saccade landing on the object. **(b)** Eye-object x-coordinate correlation for the object in (a). **(c)** An object appeared in the left visual field (LVF), with the subject making two saccades landing on the object. **(d)** Eye-object x-coordinate correlation for the object in (c).

Table S1. Summary of participant demographic characteristics. *Related to STAR Methods.*

Demographic characteristics of the 34 kindergarten children included in this study. Ancestry, race, and ethnicity were not collected as they were not relevant to the research questions.

Characteristic	Value
**Sample**	
Total participants	N = 34
**Age**	
Range	4–6 years
Mean (SD)	5.29 years (0.71)
**Sex**	
Female	18 (53%)
Male	16 (47%)
**Handedness**	
Right-handed	28 (82%)
Left-handed	5 (15%)
Bilateral	1 (3%)
**Language Background**	
German (mother tongue)	23 (68%)
Bilingual French–German	11 (32%)
**Kindergarten Grade**	
Grade 1	12 (35%)
Grade 2	22 (65%)
**Family History of Dyslexia (parent-reported)**	
No family history	26 (76%)
Positive family history	4 (12%)
Unsure	4 (12%)
**Ethics**	
Ethics approval	BASEC, University of Fribourg (No. 2023-00226)
Informed consent	Written parental consent + verbal child assent

Note: *The influence of sex on the study results was not systematically assessed due to the small sample size, which represents a limitation to the generalizability of the findings.*
